# RETRACTION: Pyrrolo[1,2‐b][1,2,5]benzothiadiazepines (PBTDs) Exert Their Anti‐proliferative Activity by Interfering With Akt–mTOR Signaling and bax:bcl‐2 Ratio Modulation in Cells From Chronic Myeloid Leukemic Patients

**DOI:** 10.1111/cas.16390

**Published:** 2024-10-30

## Abstract

**RETRACTION**: C. Di Stefano, G. Marfe, M. M. Trawinska, P. Sinibaldi‐Salimei, R. Silvestri, S. Amadori and E. Abruzzese, “Pyrrolo[1,2‐b][1,2,5]benzothiadiazepines (PBTDs) Exert Their Anti‐proliferative Activity by Interfering With Akt–mTOR Signaling and bax:bcl‐2 Ratio Modulation in Cells From Chronic Myeloid Leukemic Patients,” *Cancer Science* 101, no. 4 (2010): 991–1000, https://doi.org/10.1111/j.1349‐7006.2010.01490.x.

The above article, published online on 07 January 2010 in Wiley Online Library (wileyonlinelibrary.com), has been retracted by agreement between the journal Editor‐in‐Chief, Masanori Hatakeyama; the Japanese Cancer Association; and John Wiley & Sons Australia, Ltd. The retraction has been agreed due to several instances of duplications of western blot bands observed in Figures 3, 4, 5 and 8. Furthermore, splicing and deletion of bands was uncovered in Figures 4 and 7 respectively. Finally, western blot bands presented in Figure 4 was also found published earlier in another article. The authors provided partial raw data but the inconsistencies found could not be resolved on this basis. Due to the extent and nature of the duplications, the editors consider the results and conclusions of this study invalid.

**RETRACTION**: C. Di Stefano, G. Marfe, M. M. Trawinska, P. Sinibaldi‐Salimei, R. Silvestri, S. Amadori and E. Abruzzese, “Pyrrolo[1,2‐b][1,2,5]benzothiadiazepines (PBTDs) Exert Their Anti‐proliferative Activity by Interfering With Akt–mTOR Signaling and bax:bcl‐2 Ratio Modulation in Cells From Chronic Myeloid Leukemic Patients,” *Cancer Science* 101, no. 4 (2010): 991–1000, https://doi.org/10.1111/j.1349‐7006.2010.01490.x.

The above article, published online on 07 January 2010 in Wiley Online Library (wileyonlinelibrary.com), has been retracted by agreement between the journal Editor‐in‐Chief, Masanori Hatakeyama; the Japanese Cancer Association; and John Wiley & Sons Australia, Ltd. The retraction has been agreed due to several instances of duplications of western blot bands observed in Figures 3, 4, 5 and 8. Furthermore, splicing and deletion of bands was uncovered in Figures 4 and 7 respectively. Finally, western blot bands presented in Figure 4 was also found published earlier in another article. The authors provided partial raw data but the inconsistencies found could not be resolved on this basis. Due to the extent and nature of the duplications, the editors consider the results and conclusions of this study invalid.

